# Failure patterns after curative resection for intrahepatic cholangiocarcinoma: possible implications for postoperative radiotherapy

**DOI:** 10.1186/s12885-019-6328-3

**Published:** 2019-11-14

**Authors:** Wei Yu, Chunxiu Hu, Yongjie Shui, Kui Wu, Lili Zhang, Ying Chen, Chao Li, Jing Xu, Qichun Wei

**Affiliations:** 1 Department of Radiation Oncology, the Second Affiliated Hospital, Zhejiang University School of Medicine, Jiefang Road 88, Hangzhou, 310009 People’s Republic of China; 2 Ministry of Education Key Laboratory of Cancer Prevention and Intervention, Zhejiang University School of Medicine, Hangzhou, 310009 People’s Republic of China; 3 Department of Radiation Oncology, Zhejiang Quhua Hospital, Quzhou, 324000 People’s Republic of China; 4 Department of Radiology, the Second Affiliated Hospital, Zhejiang University School of Medicine, Hangzhou, 310009 People’s Republic of China

**Keywords:** Intrahepatic cholangiocarcinoma, Recurrence pattern, Target volume, Adjuvant radiotherapy

## Abstract

**Background:**

To explore the patterns of failures and areas at highest risk of recurrence for postoperative intrahepatic cholangiocarcinoma (IHCC), with the aim to guide IHCC adjuvant radiotherapy.

**Methods:**

Patients with IHCC who had undergone radical surgery at our institution from July 2010 to August 2017 were retrospectively analyzed. The survival and prognostic factors were analyzed by univariate and multivariate analysis. All sites of recurrence were found out and classified as the surgical margin, regional lymph nodes, liver remnant and distant metastasis. According to the recurring area at highest risk, the target volume of adjuvant radiotherapy was proposed.

**Results:**

The median follow-up time was 23.5 months (2–85 months). The median recurrence free survival (RFS) and overall survival (OS) were 12.1 months and 24.8 months, respectively. Seventy-three (73/127, 57.5%) IHCC patients developed tumor recurrence. Initial recurrences occurred in the potential postoperative radiotherapy (PORT) volume, remnant liver and distant sits were 46 (46/73, 63.0%), 36 (36/73, 49.3%) and 22 (22/73, 30.1%) cases, respectively. Of the 46 patients whose initial recurrence inside the potential PORT volume, 29 (29/73, 39.7%) developed recurrence only inside the potential PORT volume, including 13 tumor bed recurrences, 7 lymph node metastases, and 9 with both tumor bed recurrences and lymph node metastases. The most common lymph node metastases sites were nodes around the abdominal aorta, followed by lymph nodes along the celiac artery, the common hepatic artery, and in the hepatoduodenal ligament.

**Conclusions:**

High proportion of the recurrences occurred only inside the potential PORT volume, implying adjuvant radiotherapy might improve the local-regional control. Surgical margins and lymph node stations No.16a2, 9, 8, 12, 13, and 14 are suggested to be included in the radiation volume.

## Background

Intrahepatic cholangiocarcinoma (IHCC) is an uncommon neoplasm, accounting for 5–10% of all cholangiocarcinomas [1, 2]. While prior studies estimated that IHCC occupied a minority of cholangiocarcinomas (CCAs) [2], more recent assessments reveal the incidence of IHCC seems to be rising across the world [3–5]. Owing to its intrahepatic location of IHCC, early symptoms are rare and most patients present with advanced tumors [6, 7]. IHCC has a dismal survival with limited treatment options and a very high rate of recurrence or metastatis [8, 9]. The mainstay of treatment of IHCC is surgical excision, definitive roles for adjuvant chemotherapy and radiotherapy have not been found, although both are used in daily practice [10, 11].

The literature on adjuvant radiation therapy for IHCC is sparse. In the few retrospective reports found in the English literature through Pubmed, the role of postoperative radiotherapy remains controversial. Shinohara et al. analyzed 3839 patients with IHCC revealed a better median OS with surgery and adjuvant radiotherapy than surgery alone (11 months vs 6 months, *p* = 0.014) [12]. Other two small size studies also proved the value of adjuvant therapy for postoperative IHCC [13, 14]. While a meta-analysis found a non-significant improvement in overall survival with adjuvant treatment (chemotherapy, radiation or both) compared with surgery alone [15]. Another study conducted by Hammad et al. reported that adjuvant radiotherapy was associated with an improved survival for patients with R1/R2 resection, but not for those with R0 resection [16]. To date, there is no prospective randomized study concerning the benefit of adjuvant radiotherapy on IHCC patients. Study on IHCC post-operative failure pattern might help to optimize adjuvant treatment strategies. If the rate of local recurrence is high, adjuvant radiotherapy might be engaged to improve local control. On the contrary, systematic chemotherapy should be considered if distant metastases are the predominant failure patterns.

In the present study, failure patterns of 127 postoperative IHCC patients were retrospectively analyzed, with the aim of providing more reference information for the design of adjuvant treatment.

## Methods

### Patients

This study was approved by the Institutional Review Board of the Second Affiliated Hospital, Zhejiang University School of Medicine (SAHZU). From July 2010 to August 2017, 182 patients had undergone surgery for intrahepatic cholangiocarcinoma at SAHZU, histopathology diagnosis were achieved after surgery. The medical records were retrospectively reviewed, 127 radical resection patients with at least 2-month follow-up were included. Data regarding the surgical resection performed and pathologic variables including sex, age, tumor staging, T classification, N classification, tumor differentiation, resection margins, tumor size, recurrence time, tumor markers, history of hepatitis, hypertension, diabetes, bile duct stone, jaundice, fever and postoperative chemotherapy were collected. Tumor staging was performed according to the guidelines of the American Joint Committee on Cancer (AJCC) Seventh edition [17] . Follow-up period ended on February 8, 2018. The diagnosis of recurrence is mainly based on imaging findings and clinical manifestations. RFS was measured from the day of operation to tumor recurrence, and OS from the day of operation to patient’s death or last follow-up. Long-term follow-up and patient status were determined by physician consult. Specific sites of first disease recurrence, time to disease recurrence, OS and RFS were analyzed.

### Recurrence patterns

In the present study, the term “local-regional recurrence” was not used, as retroperitoneal lymph nodes recurrences are classified as distant metastases according to the 8th edition of the AJCC staging system. Instead, we use “potential volume of postoperative radiotherapy (PORT)” which including surgical margins, and high-risk lymphatic drainage area. The site(s) of initial disease recurrence, which determined from cross-sectional imaging studies (CT, MRI or PET-CT), were found out and classified as recurrences inside the potential PORT volume, residual liver, and distant recurrence. Biopsies for the recurrent lesions were encouraged. Radiologic evidence of tumor recurrence (suspicious new findings and progression of disease documented by serial imaging) was also accepted in patients who did not undergo biopsy. The date of initial disease recurrence was recorded as the time when the first suspicious radiologic finding was initially identified.

### Statistical analysis

The correlation of patient characteristics with progression-free survival and overall survival were analyzed by Kaplan Meier analysis. Patient characteristics include tumor stage, T grade, lymph node status, tumor differentiation, nerve invasion, vascular invasion, tumor size, recurrence time, tumor markers, and history of hepatitis, schistosomiasis, hypertension, diabetes, gallstones, bile duct stones, Jaundice, fever, etc. Significance was evaluated using the log-rank test. Cox proportional hazards models was used for multivariate survival analysis. Statistical significance was defined as a *p* value< 0.05. IBM SPSS Statistics 19.0 was used for statistical analyses.

## Results

### Patient characteristics

The demographics of the 127 patients are summarized in Table 1. There were 64 men (50.4%) and 63 women (49.6%). The median patient age was 58 years old (range, 26–83 years old). Preoperative tumor markers examination was done in 125 cases, elevated CA-199 and CEA were detected in 82 (67.8%) and 36 (29.8%) cases, respectively. The mean tumor size was 4.5 cm (range 1.0–11.0 cm), of which 43 (33.9%) had a tumor size ≥5 cm. The differentiation of the IHCC was as follows: poor, 42 cases (33.1%); moderate, 52 cases (40.9%); well, 15 cases (11.8%); unkwon, 18 (14.2%). Distribution according to T stage was as follows: T1, 35 cases (27.6%); T2, 56 cases (44.1%); T3, 33 cases (26.0%); and T4, 3 cases (2.4%). Lymph nodes metastasis was found in 41 of the cases (32.3%). According to the tumor TNM staging, patients had stage I, II, III, and IV disease were 28 (22.0%), 34 (26.8%), 21 (16.5%), and 44 (34.6%) cases, respectively. Twenty eight patients received adjuvant chemotherapy.

**Table 1:** Intrahepatic cholangiocarcinoma patient characteristics

Characteristics	No.	%
Gender		
Male	64	50.4
Female	63	49.6
Median age (yrs)	58	
Range (yrs)	26–83	
Node status		
N1	41	32.3
N0	86	67.7
T category		
T1	35	27.6
T2	56	44.1
T3	33	26.0
T4	3	2.4
Type of operative resection		
Less than hemihepatectomy	69	54.3
Hemihepatectomy	42	33.1
Extended hepatectomy	16	12.6
Tumor differentiation		
Well differentiated	15	11.8
Moderate differentiated	52	40.9
Poor differentiated	42	33.1
Unkown	18	14.2
Adjuvant chemotherapy	28	22.0
Tumor size, cm		
Mean	4.5	
Range	1.0–11.0	
Tumor size ≥5 cm	43	33.9
AJCC 7th Staging		
I	28	22.0
II	34	26.8
III	21	16.5
IV	44	34.6
CA199, (u/ml) > 37 U/ml	82	67.8
CEA, (ng/ml) > 5 ng/ml	36	29.8
Hepatitis	34	26.8
Hypertension	32	25.2
Diabetes mellitus	10	7.9
Bile duct stone	29	22.8
Fever	16	12.6
Jaundice	10	7.9

### Initial disease recurrence

**Fig. 1:**
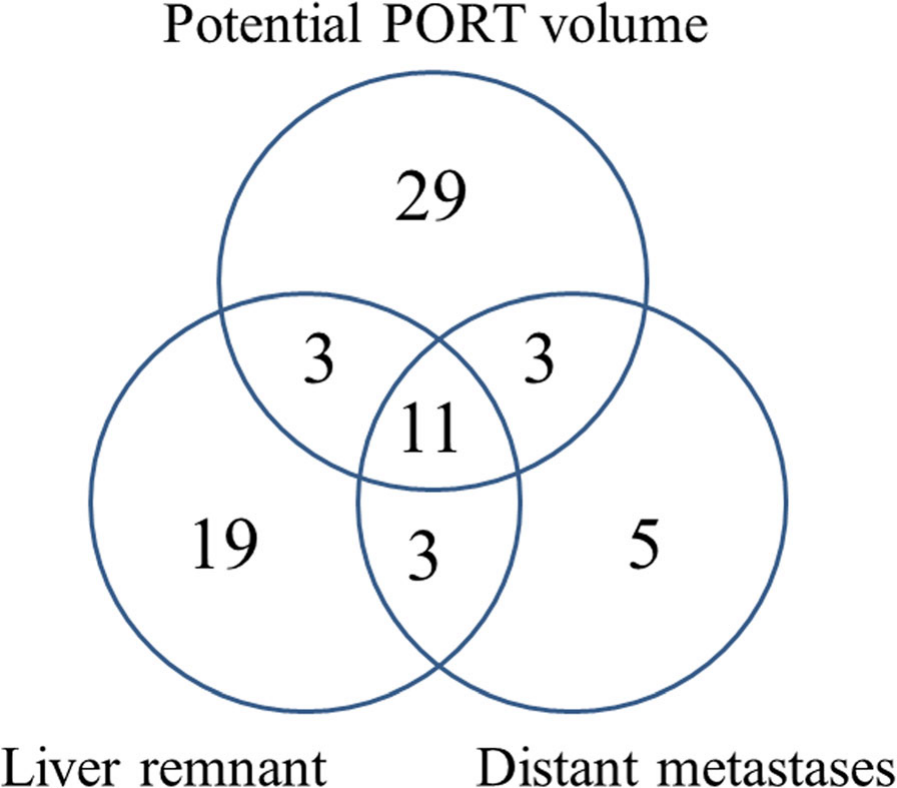
Initial recurrence pattern of intrahepatic cholangiocarcinoma (*n* = 73)

Seventy-three (73/127, 57.5%) IHCC patients developed tumor recurrence. Disease progression was mainly documented by serial imaging in 65 patients (65/73, 89.0%), and 8 patients (8/73, 11.0%) had biopsy confirmation. Table 2 and Fig. 1 show the anatomic locations of all initial tumor progressions.

**Table 2:** Recurrent patterns of intrahepatic cholangiocarcinoma

	Cases	%
Recurrences inside the potential volume of PORT	29	39.7
Surgical margin alone	13	17.8
Lymph node alone	7	9.6
Surgical margin and lymph node inside PORT volume	9	12.3
Recurrences outside the potential volume of PORT	44	60.3
Liver Remnant alone	19	26.0
Lung alone	4	5.5
Surgical margin and liver remnant	3	4.1
Surgical margin, lymph node inside and outside PORT volume	1	1.4
Surgical margin, liver remnant and lung	1	1.4
Surgical margin, lung and abdominal wall	1	1.4
Liver remnant, lymph node inside and outside PORT volume	5	6.8
Liver remnant, lymph node and adrenal	2	2.7
Liver remnant, lung and lymph node	2	2.7
Liver remnant and bone	2	2.7
Liver remnant, adrenal and bone	1	1.4
Liver remnant, lymph node, abdominal wall and peritoneum	1	1.4
Lymph node and bone	1	1.4
Peritoneum alone	1	1.4
Total no. of patients with recurrence	73	100.0

Initial recurrences occurred in the potential PORT volume, remnant liver and distant sits were 46 (46/73, 63.0%), 36 (36/73, 49.3%) and 22 (22/73, 30.1%) cases, respectively. Among them, 20 cases had multiple sites of initial disease recurrence. Fifty-four patients had no evidence of disease at the last follow-up.

Of the 46 patients whose initial recurrence inside the potential PORT volume, 29 (29/73, 39.7%) developed recurrence only inside the potential PORT volume, including 13 tumor bed recurrences, 7 lymph node metastases, and 9 with both tumor bed recurrences and lymph node metastases. Three had synchronous recurrences at remnant liver, another 3 had synchronous recurrences at distant sites, and 11 had synchronous recurrences at remnant liver and distant sites.

Thirty-six patients developed initial recurrence in the remnant liver. Among them, 19 had remnant liver lesions as the only recurrences, 3 had synchronous recurrences in the potential PORT volume, another 3 had synchronous recurrences at distant sites, and 11 had synchronous recurrences at distant sites and the potential PORT volume.

Twenty-two had distant metastases at initial recurrence, 5 of them were distant metastases only (4 lung, 1 peritoneum), 3 had synchronous recurrences in the potential PORT volume, another 3 had synchronous remnant liver recurrences, and 11 had synchronous recurrences in the potential PORT volume and remnant liver.

Among the patients who received adjuvant chemotherapy, 17 patients relapsed. Seven patients relapsed inside the PORT volume, 7 patients relapsed outside the PORT volume, 3 patients relapsed both inside and outside the potential volume. The recurrence pattern was similar between patients received adjuvant therapy and the whole group.

The common sites of lymph node metastases were lymph nodes around the abdominal aorta (station No.16, *n* = 18), lymph nodes along the celiac artery (No.9, *n* = 13), lymph nodes along the common hepatic artery (No.8, *n* = 11), lymph nodes in the hepatoduodenal ligament (No. 12, *n* = 8), lymph nodes on the posterior aspect of the pancreatic head (No.13, *n* = 4), lymph nodes at the root of the mesenterium (No.14, *n* = 2). All patients with station No.16 recurrences had metastatic lesions at other lymph node stations or distant sites. Among them, 11 cases had lesions in No.16a2 with no metastases in No.16b1, 6 had lesions in No.16a2 and No.16b1. Only 1 case had lymph node metastases at station No.16b1 without lesion at No.16a2, but they had multiple metastatic nodes on the posterior aspect of the pancreatic head.

### Follow-up and survival

**Fig. 2:**
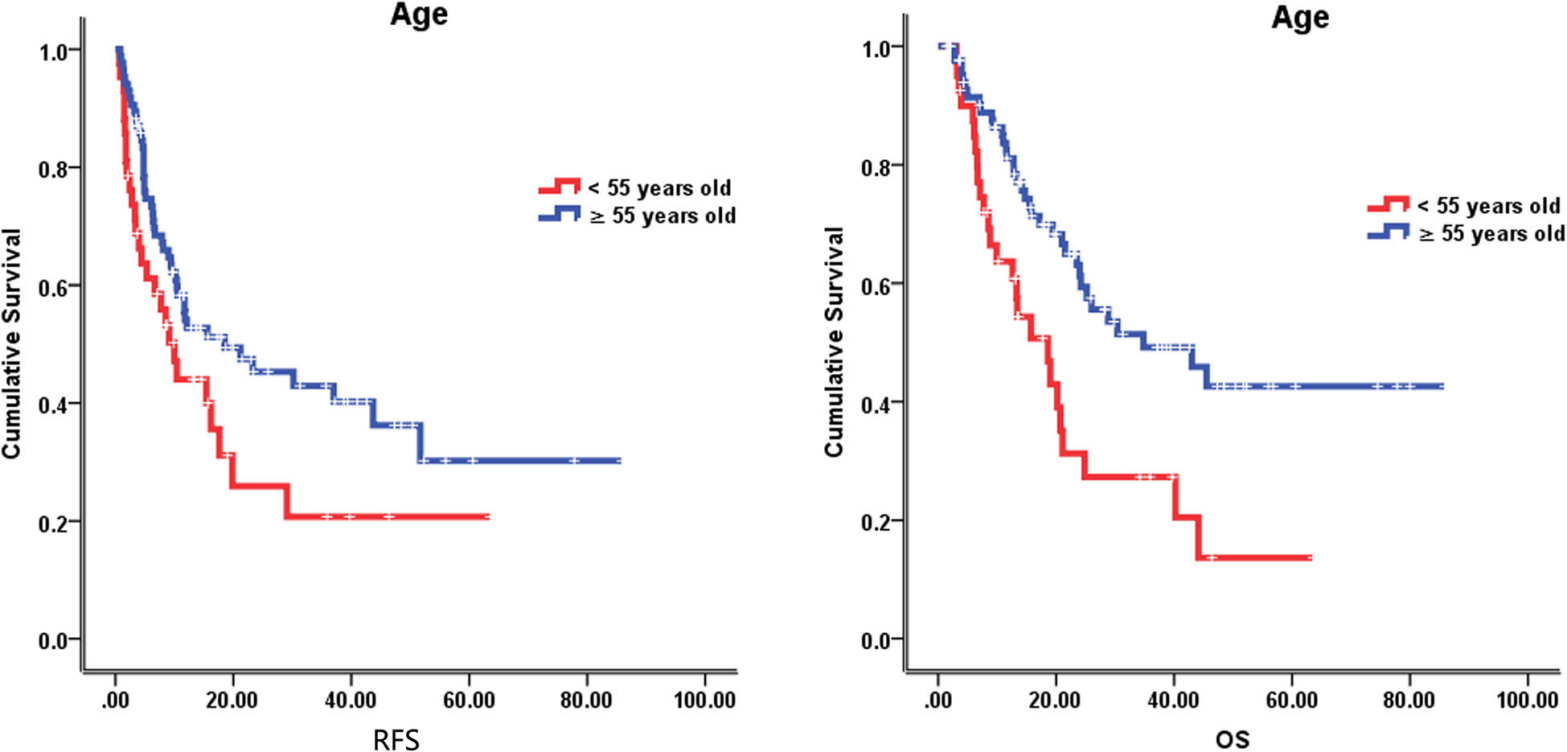
Prognostic value of age in IHCC. Kaplan–Meier curves of RFS and OS in group with age less than 55 years old and equal to or more than 55 years old

**Fig. 3:**
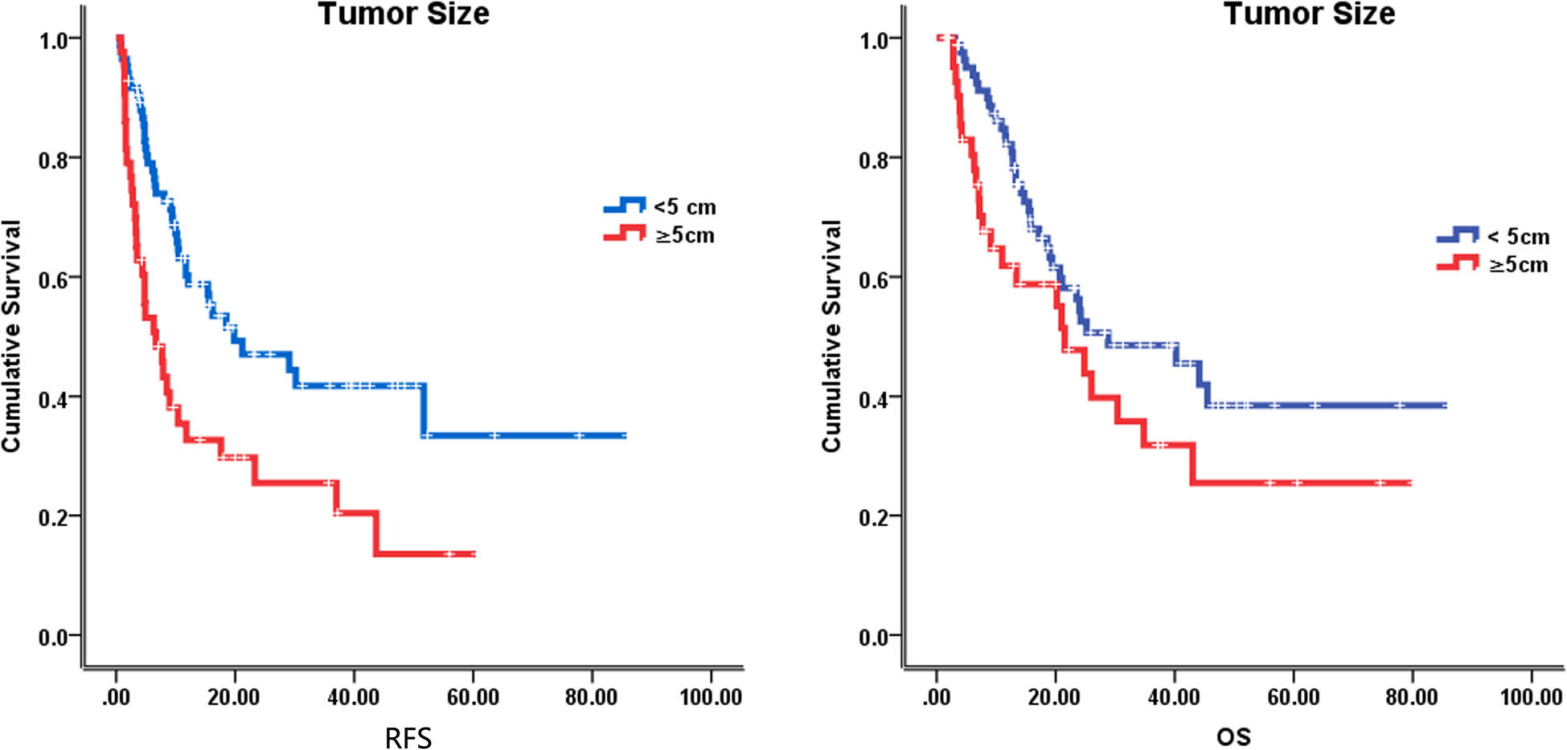
Prognostic value of tumor size in IHCC. Kaplan–Meier curves of RFS and OS in group with tumor size less than 5 cm and equal to or more than 5 cm

**Fig. 4:**
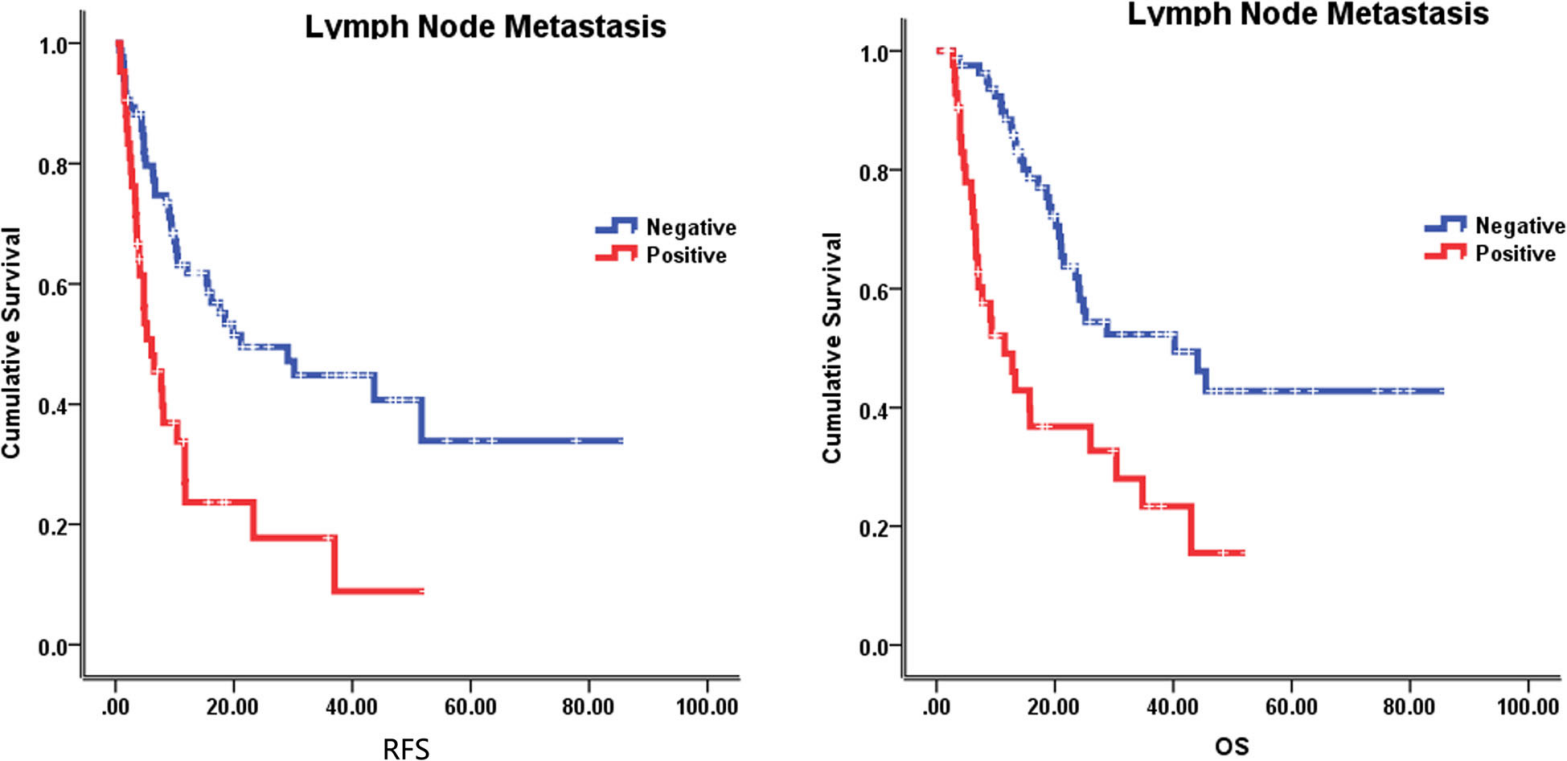
Prognostic value of lymph node metastasis in IHCC. Kaplan–Meier curves of RFS and OS in group with lymph node metastasis and without lymph node metastasis

**Fig. 5:**
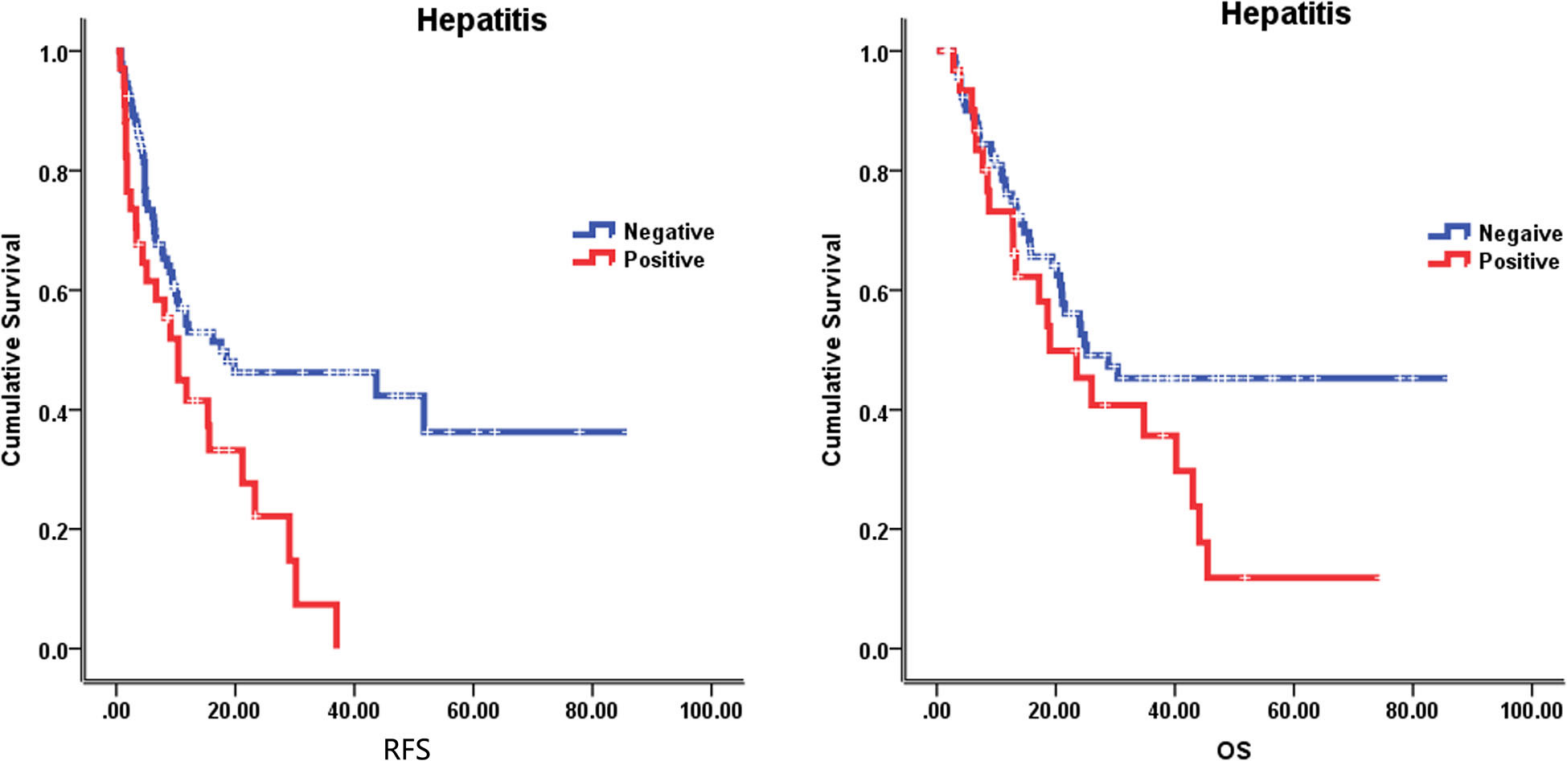
Prognostic value of hepatitis in IHCC. Kaplan–Meier curves of RFS and OS in group with hepatitis and without hepatitis

**Fig. 6:**
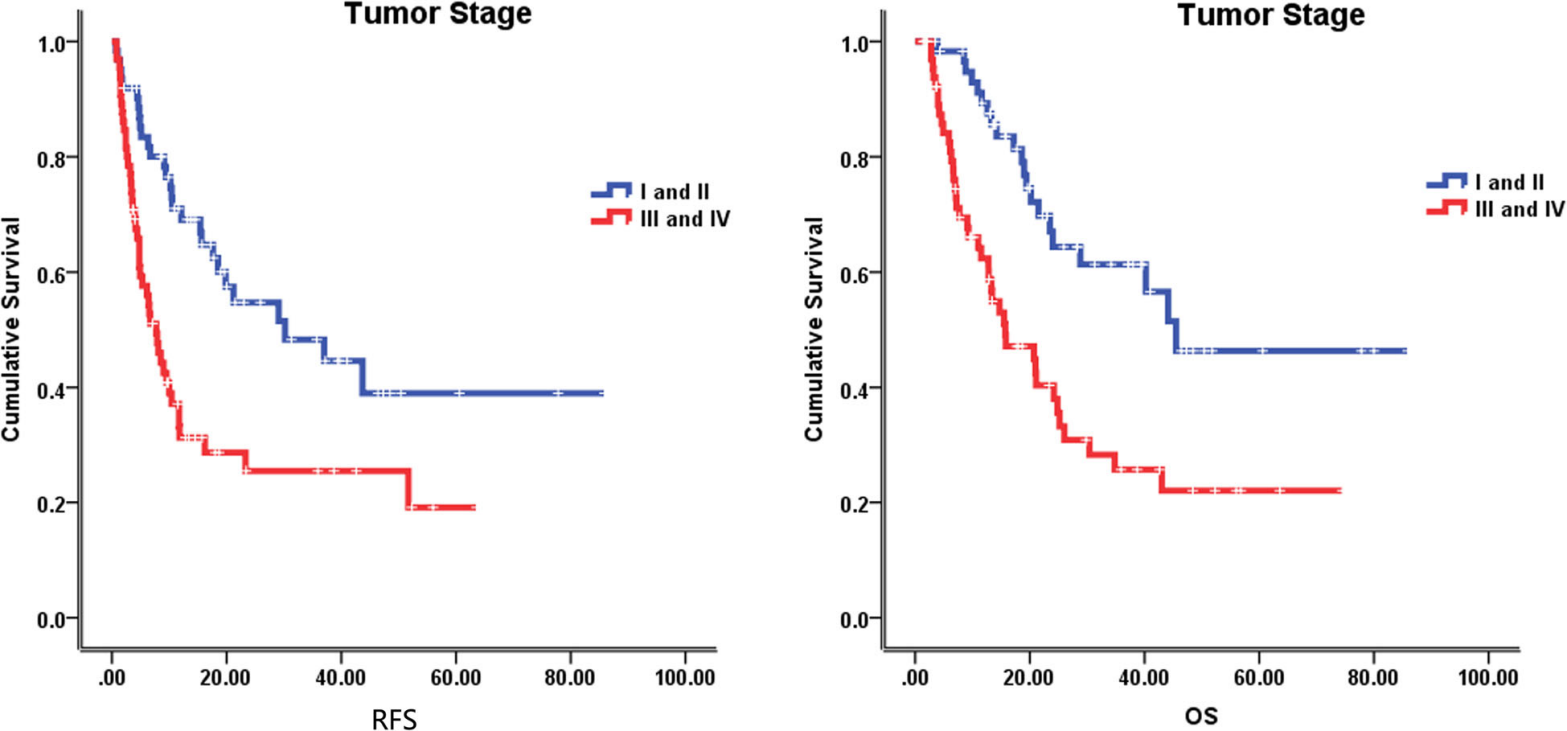
Prognostic value of tumor stage in IHCC. Kaplan–Meier curves of RFS and OS in group with tumor stage I, II and tumor stage III, IV

**Fig. 7:**
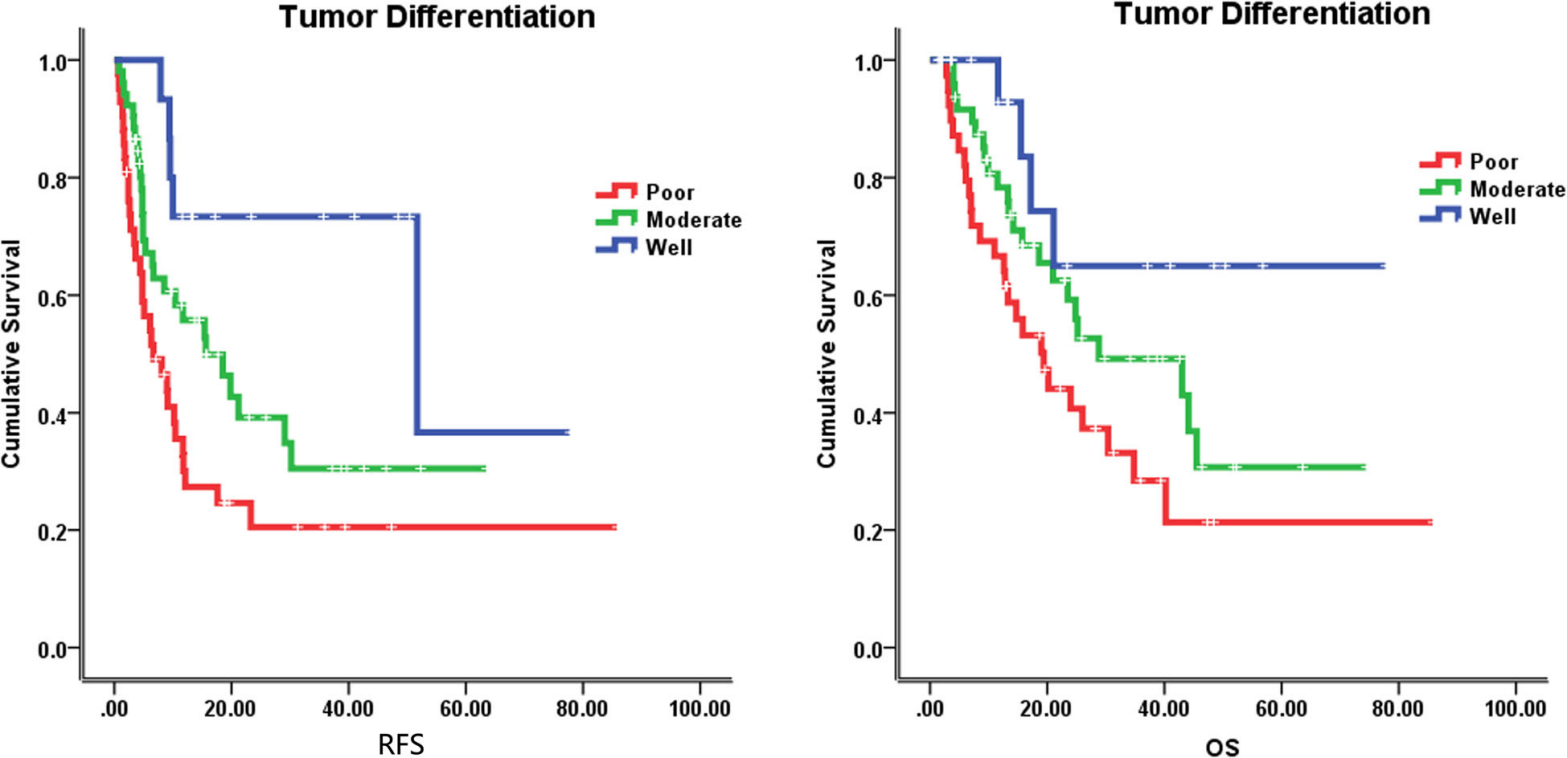
Prognostic value of tumor differntiation in IHCC. Kaplan–Meier curves of RFS and OS in group with well, moderate and poor tumor differentiation

The median follow-up time was 23.5 months (2–85 months), at the last follow-up on February 8, 2018, 59 patients were alive. The median recurrence free survival (RFS) and overall survival (OS) were 12.1 months and 24.8 months, respectively. In the univariate analysis of the entire cohort, improved survival was associated with age ≥ 55 years (RFS, 18.5 vs 10.0 months, *p* = 0.046; OS, 34.8 vs 18.6 months, *p* = 0.002) (Fig. 2), tumor size < 5 cm (RFS, 19.8 vs 6.7 months, *p* = 0.001; OS, 28.8 vs 21.5 months, *p* = 0.071) (Fig. 3), without lymph node metastasis (RFS, 21.2 vs 6.1 months, *p* < 0.001; OS, 40.2 vs 11.5 months, *p* < 0.001) (Fig. 4), without hepatitis (RFS, 17.6 vs 10.4 months, *p* = 0.005; OS, 25.2 vs 19.0 months, *p* = 0.079) (Fig. 5), early tumor staging (stage I and II vs stage III and IV, RFS, 30.2 vs 7.7 months, *p* < 0.001; OS, 45.5 vs 15.7 months, *p* < 0.001) (Fig. 6) and better tumor differentiation (poor vs moderate vs well, RFS, 6.7 vs 15.6 vs 51.7 months, *p* = 0.027; OS, 19.4 vs 28.8 months, not reached for well differentiation patients, *p* = 0.003) (Fig. 7). The results of the univariate analysis are summarized in Table 3. In the multivariate analysis, RFS of tumor size (RR 2.191; 95% confidence interval [CI] 1.257–3.817; *p* = 0.003) and tumor differentiation (RR 0.621; 95% confidence interval [CI] 0.408–0.947; *p* = 0.027) maintained significance. OS of age (RR 0.418; 95% confidence interval [CI] 0.236–0.740; *p* = 0.003) maintained significance.

**Table 3:** Univariate analyses for PFS and OS among 127 patients who underwent curative surgery for IHCC

	Median survival (month)	*P* value	Median survival (month)	*P* value
Age				
< 55 years old	10.0		18.6	
≥ 55 years old	18.5	0.046	34.8	0.002
Sex				
Male	15.6		28.8	
Female	10.5	0.778	21.0	0.599
TMN staging				
I and II	30.2		45.5	
III and IV	7.7	< 0.001	15.7	< 0.001
T classification				
I	15.4		23.5	
II	10.4		40.2	
III	16.2		24.2	
IV	4.0	0.061	Not reached	0.948
Lymph node metastasis				
N0	21.2		40.2	
N1	6.1	< 0.001	11.5	< 0.001
Tumor differentiation				
Well	51.7		Not reached	
Moderate	15.6		28.8	
Poor	6.7	0.003	19.4	0.027
Resection Margin				
Positive	4.8		21.0	
Negative	15.6	0.020	25.2	0.304
Tumor size				
< 5 cm	19.8		28.8	
≥ 5 cm	6.7	0.001	21.5	0.071
Hepatitis				
Positive	10.4		19.0	
Negative	17.6	0.005	25.2	0.079
Hypertension				
Yes	11.7		21.1	
No	15.6	0.856	43.0	0.074
Bile duct stone				
Yes	11.7		18.6	
No	15.6	0.657	30.4	0.063
Jaundice				
Yes	10.2		19.4	
No	15.4	0.406	25.2	0.386
Fever				
Yes	10.5		21.1	
No	15.4	0.737	24.8	0.920
Postoperative chemotherapy				
Yes	11.7		43.0	
No	15.6	0.939	23.5	0.170
CA-199				
Elevated	15.4		26.0	
Not elevated	11.7	0.961	28.8	0.887
CEA				
Elevated	7.7		15.5	
Not elevated	17.6	0.183	40.2	0.016

## Discussion

This study analyzed the failure patterns of IHCC after curative resection. Tumor recurrences were found in up to 60% of the patients. Failures inside the potential PORT volume were found to be the most common sites of initial recurrences, followed by metastases in remnant liver and distant sits. Our findings implicate the possible benefits of postoperative radiotherapy for IHCC patients.

The frequency of postoperative failures in IHCC, including local-regional recurrence and remnant liver dissemination or distant metastases, has been reported to range from 53.5 to 80.0% [18–22]. Our result, with 57.5% of IHCC patients developed postoperative recurrence, was in line with the previous reports. For radiation oncologists, data on the specific patterns of recurrence can help to guide postoperative therapeutic approaches. According to the study by Hyder et al., the most common recurrent site after surgery of IHCC was intrahepatic [21]. While Doussot et al. reported a time dependent recurrence pattern. Recurrence within 24 months most often involved the liver (82.7%), and the most recurrences after 24 months were extrahepatic (61.1%) [22]. Recently, high local-regional recurrent rates as 62.5 and 68% of the total failures have been reported [18, 19]. The reported proportion of local-regional recurrence varies. Different statistical methods may explain for the inconsistent results. For example, margin recurrences usually be regarded as local failures, and might also be included in the intrahepatic failures. Furthermore, results could differ from whether initial recurrences or cumulative events are counted. In our study, recurrences in the potential PORT volume, i.e., surgical margins recurrence, regional and retroperitoneal lymph node metastasis, were found to be the most common relapse sites, accounting for 63% of the initial failures, which is consistent with the results of Song [18] and Luvira [19]. Chen et al. analyzed 320 surgical cases with clinically negative lymph node (T1–3N0M0) IHCC and observed 76 cases occurred lymph node metastasis (LNM) (76/320, 23.8%). And histological differentiation as well as tumor boundary and tumor size significantly correlated with LNM [23]. Postoperative local-regional recurrence for IHCC seems to be common, which imply the potential benefit of adjuvant radiotherapy, especially for those with high risk factors.

It is worthwhile pointing out that, up to 40% of the recurrences in our patient series occurred only inside the potential PORT volume, without lesions in remnant liver and distant sits. Similar figures could be found in the literature. In the report by Song et al. [18], the incidence of local-regional recurrence only as the first site of failure was 33%, which was the dominant pattern of failure for their patient series. Moreover, additional 18 (18/66, 27.3%) of their patients had non-regional lymph node metastases. Majority of such non-regional nodes could be inside the potential PORT volume, together with the above mentioned 33% of the cases, the proportion of recurrence only inside the radiation volume could be around 50%. In another study by Luvira et al. [19], recurrences only at surgical margin and regional lymph node account for 27.5% of the total failures. However, 15% patients had aortocaval regional lymph node metastases alone or with surgical margin and regional lymph node metastases. So, totally 42.5% of the patients had recurrences confined to the potential PORT volume. Taken together, about 40% of the IHCC patients recur strictly inside the potential PORT volume as the first site of failure. These findings suggest adjuvant radiotherapy might improve the local-regional control.

**Fig. 8:**
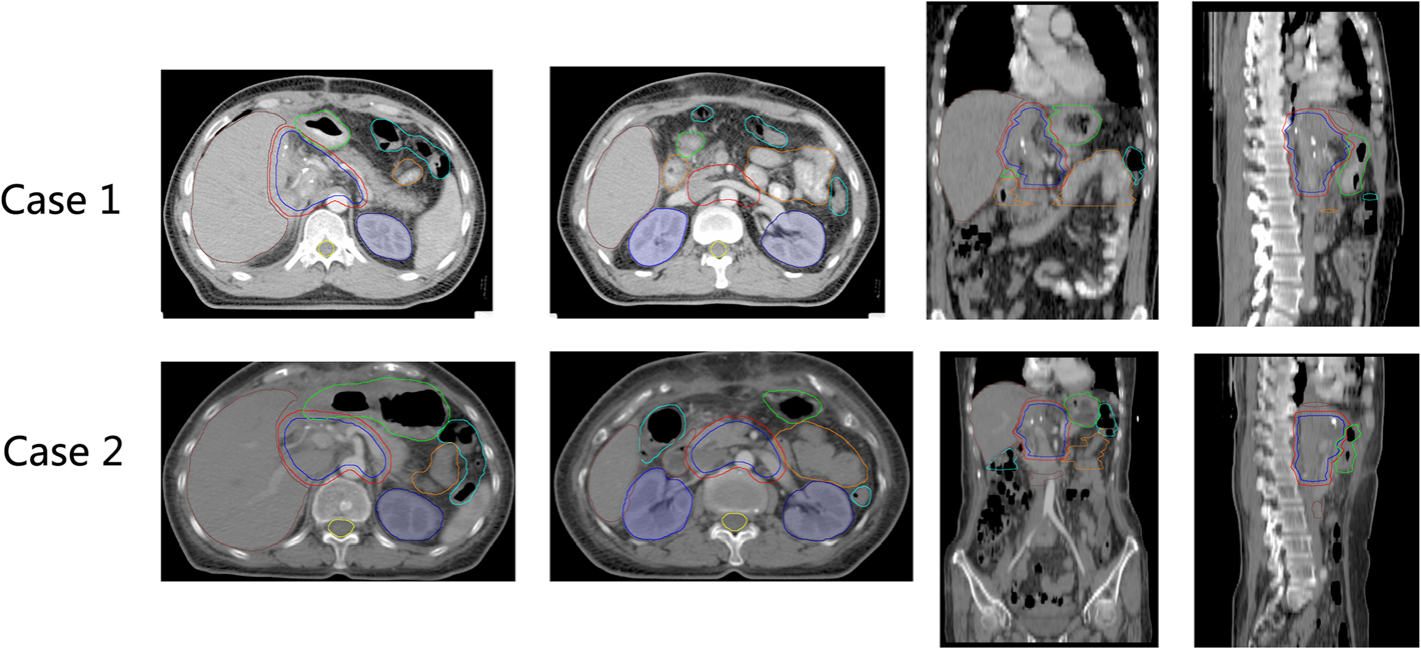
The potential PORT volume in 2 simulated cases. CTV: blue line, PTV: red line, Axial, coronal and sagittal views are shown for case 1 and case 2

The target volume of adjuvant radiotherapy was proposed basing on the local recurrence pattern, mainly includes the tumor bed, and the corresponding high-risk lymphatic drainage area. According to our findings, the most common sites of lymph node metastases were stations No.16, 9, 8, 12, 13, and 14, these lymphatic drainage areas were suggested to be in the radiation volume (Fig. 8). As station No.16b1 metastases rarely recurred isolatedly, they relapsed as a consequent of lymph flow reflux after the metastases in the above mentioned node stations. We speculate that station No.16b1 metastases might be prevented or reduced if the area inside the proposed radiation volume is under well control. So, it might be suitable not to encompass station No.16b1 in the potential PORT volume. Since lymphatic drainage areas classified as distant metastases were included in the proposed radiation volume, the term “potential volume of PORT” was used instead of “local-regional recurrence”.

In our study, 44 patients developed recurrences outside the potential volume of PORT. For these patients, chemotherapy may be helpful. Until now, the role of chemotherapy in IHCC is still controversial. Miura et al. reported that there was no difference in the median OS between the observation group and the chemotherapy group after resection of intrahepatic cholangiocarcinoma (23 vs 20 months, *P* = 0.09) [24]. Edeline et al. conducted a phase III multicenter randomized controlled clinical trial of gemcitabine plus oxaliplatin in patients with intrahepatic cholangiocarcinoma after operation. One hundred ninety-six patients with intrahepatic cholangiocarcinoma were randomly divided into gemcitabine plus oxaliplatin group and observation group. They found longer RFS and OS in gemcitabine plus oxaliplatin group, but there was no statistical difference. In the chemotherapy group, grade III-IV side effects increased [25]. Schweitzer et al. divided 25 pairs of patients with intrahepatic cholangiocarcinoma after operation into adjuvant chemotherapy group and non-adjuvant chemotherapy group. The results showed that the total survival time of adjuvant chemotherapy group was significantly longer than that of non-adjuvant chemotherapy group [26]. Reames retrospectively analyzed 1154 patients with intrahepatic cholangiocarcinoma after radical resection, 347 (30%) received adjuvant chemotherapy, 184 of whom received gemcitabine-based chemotherapy. The results showed that chemotherapy did not change the prognosis of all patients with intrahepatic cholangiocarcinoma, but the subgroup with high risk of recurrence can benefit from chemotherapy [27]. A randomised, controlled, multicentre, phase 3 study (BILCAP) compared capecitabine with observation in resected biliary tract cancer and reported that capecitabine can improve overall survival in patients with resected biliary tract cancer when used as adjuvant chemotherapy [28]. Chemotherapy is not recommended as an adjuvant treatment for intrahepatic cholangiocarcinoma in this year’s NCCN guidelines. While this year’s ASCO clinical practice guidelines recommend capecitabine for 6 months after surgery for intrahepatic cholangiocarcinoma.

In this study, we found patients younger than 55 years old, tumor size larger than 5 cm, with later TNM stage, lymph node metastasis, and hepatitis were associated with poorer outcome. In previous reports, several prognostic factors were identified for IHCC patients, including tumor size, lymph node metastasis, TNM stage and tumor differentiation [29–33]. Our results were in accordance with the previous studies. As regards to the prognostic value of patient age and hepatitis history, controversial results were reported. In a retrospective study by Zhang et al. [34], in line with our result, hepatitis was found to be with poor prognosis in IHCC, while in the study by Zhou et al., hepatitis was a favorable prognostic factor [35]. The prognositic value of age was controversial, most study reported that age had no prognostic value in cholangiocarcinoma [34, 36–38]. Mavros et al. and Kato et al. found that older age was associated with worse prognosis [39, 40]. However, in our study, we found that patients with age < 55 years old had shorter RFS and OS than those with age ≥ 55 years old, which was in accordance with the results reported by Yamada et al. [41] Our data added some information in this respect, and the prognositic value of age is still uncertain.

## Conclusions

This study analyzed the failure patterns after curative resection for intrahepatic cholangiocarcinoma. About two thirds of IHCC would recur after operation, with 60% of recurrences inside the potential volume of PORT. Importantly, up to 40% of the recurrences occurred only inside the potential PORT volume, without lesions in remnant liver and distant sits. Surgical margins and lymph node stations No.16a2, 9, 8, 12, 13, and 14 were the most common sites of recurrence, and should be included in the radiation volume. Our results implicate the possible benefits of postoperative radiotherapy for intrahepatic cholangiocarcinoma patients. As IHCC is a low incidence tumor type, prospective multicenter single arm clinical trial with radiation involved in the adjuvant therapies might be feasible.

## Data Availability

Datasets can be retrieved from authors by formal request from interested readers. Datasets will not be directly shared on public link as the national personal data protection act.

## Abbreviations

AJCC: American Joint Committee on Cancer
CCAs: Cholangiocarcinomas
CI: Confidence interval
IHCC: Intrahepatic cholangiocarcinoma
LNM: Lymph node metastasis
OS: Overall survival
PORT: Postoperative radiotherapy
RFS: Recurrence free survival
RR: Risk ratio
SAHZU: Zhejiang University School of Medicine
SCI: Science Citation Index
